# Definitive chemoradiotherapy *versus* neoadjuvant chemoradiotherapy followed by radical surgery for locally advanced oesophageal squamous cell carcinoma: meta-analysis

**DOI:** 10.1093/bjsopen/zrac125

**Published:** 2022-12-07

**Authors:** Sivesh K Kamarajah, Richard P T Evans, Ewen A Griffiths, James A Gossage, Philip H Pucher

**Affiliations:** Department of Upper Gastrointestinal Surgery, Queen Elizabeth Hospital Birmingham, University Hospitals Birmingham NHS Foundation Trust, Birmingham, UK; Institute of Cancer and Genomic Sciences, College of Medical and Dental Sciences, University of Birmingham, Birmingham, UK; Department of Upper Gastrointestinal Surgery, Queen Elizabeth Hospital Birmingham, University Hospitals Birmingham NHS Foundation Trust, Birmingham, UK; Institute of Cancer and Genomic Sciences, College of Medical and Dental Sciences, University of Birmingham, Birmingham, UK; Department of Upper Gastrointestinal Surgery, Queen Elizabeth Hospital Birmingham, University Hospitals Birmingham NHS Foundation Trust, Birmingham, UK; Institute of Cancer and Genomic Sciences, College of Medical and Dental Sciences, University of Birmingham, Birmingham, UK; Department of Surgery, St. Thomas’ Hospital, London, UK; Department of Surgery, Portsmouth Hospitals University NHS Trust, Portsmouth, UK; Division of Surgery, Imperial College London, London, UK

## Abstract

**Background:**

The literature lacks robust evidence comparing definitive chemoradiotherapy (dCRT) with neoadjuvant chemoradiotherapy and surgery (nCRS) for oesophageal squamous cell carcinoma (ESCC). This study aimed to compare long-term survival of these approaches in patients with ESCC.

**Methods:**

A systematic review performed according to PRISMA guidelines included studies identified from PubMed, Scopus, and Cochrane CENTRAL databases up to July 2021 comparing outcomes between dCRT and nCRS for ESCC. The main outcome measure was overall survival (OS), secondary outcome was disease-free survival (DFS). A meta-analysis was conducted using random-effects modelling to determine pooled adjusted multivariable hazard ratios (HRs).

**Results:**

Ten studies including 14 092 patients were included, of which 30 per cent received nCRS. Three studies were randomized clinical trials (RCTs) and the remainder were retrospective cohort studies. dCRT and nCRS regimens were reported in six studies and surgical quality control was reported in two studies. Outcomes for OS and DFS were reported in eight and three studies respectively. Following meta-analysis, nCRS demonstrated significantly longer OS (HR 0.68, 95 per cent c.i. 0.54 to 0.87, *P* < 0.001) and DFS (HR 0.50, 95 per cent c.i. 0.36 to 0.70, *P* < 0.001) compared with dCRT.

**Conclusion:**

Neoadjuvant chemoradiotherapy followed by oesophagectomy correlated with improved survival compared with definitive chemoradiation in the treatment of ESCC; however, there is a lack of literature on RCTs.

## Teaser

Neoadjuvant chemoradiotherapy followed by oesophagectomy may offer superior overall and disease-free survival compared with definitive chemoradiation in the treatment of oesophageal squamous cell carcinoma; however, definitive prospective trial data are required.

## Introduction

Current national guidelines recommend that patients with locally advanced oesophageal squamous cell carcinoma (ESCC) be offered either definitive chemoradiotherapy (dCRT), or neoadjuvant chemoradiotherapy followed by radical surgery (nCRS)^1,2^; however, the absence of clear guidance in favour of one treatment modality over another has led to significant variation in radical treatment strategies^1,3,4^. This lack of clear evidence is reflected in at least one study highlighting the varied opinions regarding optimal treatment and a lack of either unified opinion or equipoise among surgeons and oncologists^5^.

While both dCRT and nCRS have been recognized as valid treatments for locally advanced ESCC^1,6–11^, high-quality evidence supporting either treatment strategy over the other remains limited. Current national guidelines, including the National Comprehensive Cancer Network in the USA^1^ and the National Institute for Health and Care Excellence in the UK^2^, as well as existing meta-analyses and a Cochrane review, draw exclusively upon the limited data from the only two randomized clinical trials (RCTs)^12,13^ comparing dCRT with nCRS. These failed to show any significant survival difference between the two treatment modalities, whereas more recent large-scale cohort studies have called this equipoise into question.

Despite ongoing interest in this topic, the quality of the majority of published data from cohort studies on this topic remains weak due to the high risk of bias from lack adjustment for relevant prognostic factors and the lack of stratified reporting of outcomes for patients with ESCC. Many studies group ESCC together with adenocarcinoma to represent oesophageal cancer as a whole, despite increasing recognition of the differences in tumour biology between the two histological subtypes, and differing responsiveness to treatments such as radiotherapy^6^.

Owing to the lack of clarity on comparative outcomes for the two predominant treatment strategies for ESCC, this study aims to perform a systematic review and meta-analysis of dCRT *versus* nCRS in patients with locally advanced ESCC.

## Methods

### Search strategy

A systematic review was performed according to the PRISMA Guidelines^14^. A systematic and comprehensive search was undertaken of MEDLINE, Embase, and Cochrane Library databases, searching for studies published until 23 July 2021. Searches of databases included the following terms: ‘cancer of esophagus’ or ‘esophageal cancer’ and ‘chemoradiotherapy’ and ‘oesophagectomy’ or ‘esophagectomy’. The full search strategy with all the included search terms is presented in *Table S1*. This systematic review and meta-analysis were prospectively registered on PROSPERO (​​CRD42021275438).

### Inclusion and exclusion criteria

Inclusion criteria were: comparative studies that reported differences in patient survival after treatment for locally advanced (non-metastatic) ESCC with either dCRT or nCRS; studies communicated in the English language; studies published between 2001 and 2020 inclusive; and high-quality studies as defined by criteria detailed below. Exclusion criteria were: studies that included patients with non-malignant indications for surgery or gastric malignancies; no stratification survival outcomes by patients with ESCC; non-comparative studies only reporting outcomes; studies not reporting overall survival (OS) or disease-free survival (DFS); case reports (fewer than five patients), review articles, conference abstracts; and studies including other operations and/or no surgical treatment.

After the primary literature search, three independent researchers screened the remaining titles and abstracts. Of the papers considered for inclusion, the full text was reviewed. When consensus on the inclusion of a study could not be reached, discrepancies were resolved by consulting the three senior researchers. Where multiple studies analysed the same data set or population, the most recent paper was selected unless different outcomes were reported.

### Study outcomes

The primary outcome was OS, with secondary outcomes of DFS, as well as surgical outcomes in patients receiving nCRS. DFS was defined as the time from surgery to first recurrence or last known follow-up.

### Data extraction

Three researchers extracted the following data from the included studies: first author, year of publication, study interval, type of study design, number of patients, and geographical region. The reported patient characteristics were: age, BMI, sex, tumour site, anastomotic level and type, operation method (transthoracic, McKeown, minimally invasive, or transhiatal), and reported surgical outcomes in patients undergoing nCRS.

### Assessment of study quality

**Fig. 1.**
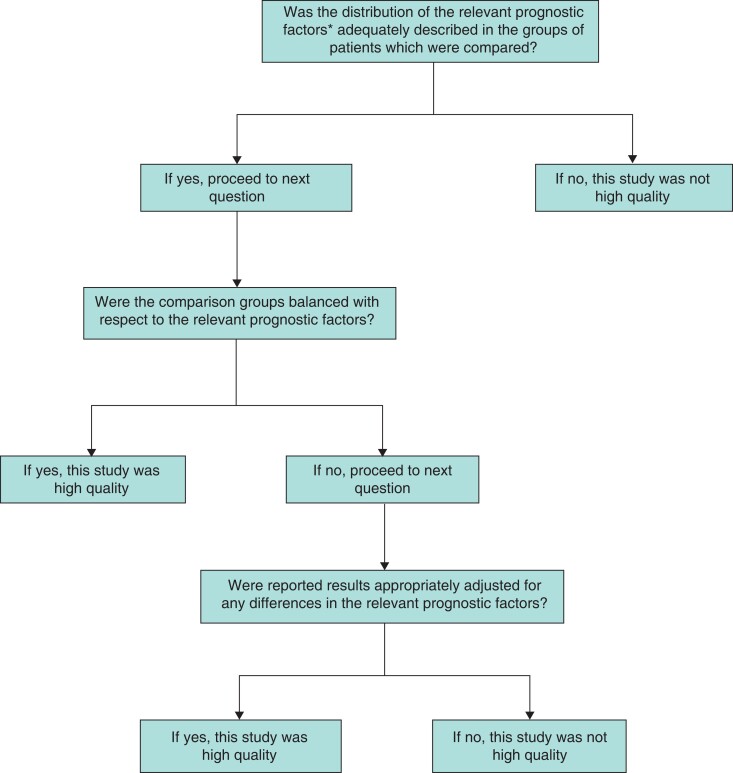
Quality assessment flow chart

Two researchers independently appraised the methodological quality and standard of outcome reporting of the included studies, with any discrepancies resolved through discussion among themselves or in consultation with the senior researchers. Studies were reviewed to assess evidence of control for major prognostic factors to assess risk of bias to determine the quality of studies, as shown in *Fig. 1*^15,16^. Only studies meeting these criteria were included for subsequent meta-analysis. The quality of the included studies was assessed using the Risk of Bias In Non-randomized Studies of Exposures (ROBINS-E)^17^ and the Cochrane Risk of Bias for RCTs^18^.

### Statistical analysis

This systematic review and meta-analysis was conducted in accordance with the recommendations of the Cochrane Library and PRISMA Guidelines^14,19^. Data analysis was undertaken using R Foundation Statistical software (R 3.2.1; R Foundation for Statistical Computing, Vienna, Austria) as previously described, to produce a random-effects meta-analysis for each outcome, providing pooled odds ratios (ORs) with 95 per cent confidence intervals (c.i.). The *I*^2^ test was used to evaluate statistical heterogeneity of the included studies, with levels of heterogeneity defined as not important (*I*^2^ 0–40 per cent), moderate (*I*^2^ 30–60 per cent), substantial (*I*^2^ 50–90 per cent), or considerable (*I*^2^ 75–100 per cent)^20^. The chi-squared test was used for the same purpose, with a statistical significance level of *P* < 0.050 indicating the presence of statistical heterogeneity.

## Results

### Study characteristics

**Fig. 2.**
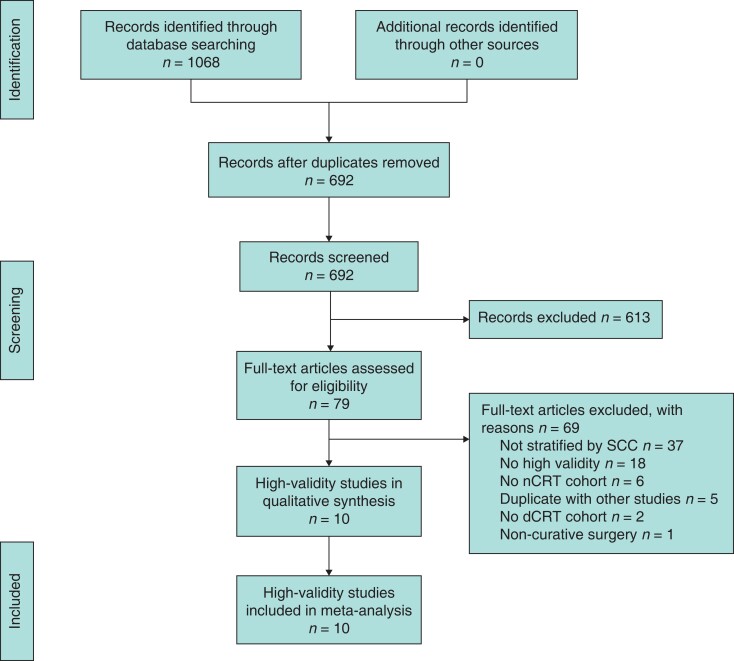
dCRT, definitive chemoradiotherapy; nCRT, neoadjuvant chemoradiotherapy and surgery; SCC, squamous cell carcinoma

Of the 1068 studies identified from the literature search, 79 underwent full text review, of which 10^12,13,21–28^ were included in the systematic review and meta-analysis. Overall, 69 studies were excluded because of not having stratified outcomes by patients with ESCC (37 studies), not having high validity (*n* = 18), no inclusion of an nCRS cohort (six studies), no inclusion of a dCRT cohort (two studies), duplication with published studies (five studies), and inclusion of non-curative surgery (one study). Reasons for exclusion are presented in *Table S2*. Ten studies comprising 12 132 patients were included in the systematic review and meta-analysis as presented in *Fig. 2*. Study, patient-, and tumour-level characteristics of included studies are presented in *Table 1*. Studies identified were from Asia (four studies), Europe (three studies), North America (two studies), and South America (one study). All studies were either retrospective cohort studies (seven studies) or RCTs (three studies). The overall risk scores of the cohort studies according to ROBINS-E and RCTs according to the Cochrane Risk of Bias are reported in *Tables S3* and *S4* respectively.

**Table 1:** Study and patient characteristics of included studies in the systematic review

Study name	Duration	Study country	Centres	Patients, (*n*)	Age, years (median [range])	Male, (%)	Clinical stage III, (%)
**Randomized clinical trials**							
Stahl 2005^12^	1994–2002	Germany	Multiple	172	57.0 (36.0–71.0)	80.2	16.9
Bedenne 2007^13^	1993–2000	France	Single	259	57.3 (9.2)*	93.4	NR
Park 2019^28^	2012–2016	Korea	Single centre	37	60.0 (55.0–67.0)	96.3	54.1
**Cohort studies**							
Lee 2003^21^	1993–1996	Korea	Single centre	88	63.0 (42.0–81.0)	93.2	27.3
Liu 2017^22^	2002–2012	Taiwan	Single centre	182	NR	78.0	75.8
Barbetta 2018^23^	2000–2016	USA	Single	232	NR	54.3	66.0
Sheil 2018^24^	2000–2014	Ireland	Single centre	222	NR	NR	NR
Wang 2019^25^	2008–2014	Taiwan	Multiple registry	5832	NR	94.8	81.8
Duarte 2020^26^	2000–2013	Brazil	Multiple registry	1347	NR	84.9	38.4
Kamarajah 2020^27^	2004–2015	USA	Multiple NCDB	5621	NR	64.1	NR

Values are median unless otherwise indicated.

*mean (SD). NR, not reported; NCDB, National Cancer Database.

### Patient characteristics

The review included 14 092 patients, of which 64 per cent (8602 patients) received dCRT and 36 per cent (*n* = 4877) received nCRS for ESCC. Tumour location was reported in six studies (12132 patients) and rates of proximal, middle, and distal cancers were 22 per cent (*n* = 2619), 37 per cent (*n* = 4497), and 30 per cent (*n* = 3689) respectively; however, tumour location between both groups was reported in six studies (dCRT, *n* = 7519; and nCRS, *n* = 4525). Rates of tumour location between dCRT and nCRS were similar for distal (15 per cent *versus* 15 per cent) cancers, but higher with dCRT than nCRS for proximal (18 per cent *versus* 3 per cent), and middle (21 per cent *versus* 15 per cent) cancers. The majority of studies included patients with stage II–III ESCC (six studies), followed by stage I–III (*n* = 2), stage I–IVA (*n* = 1), and stage II–IV (*n* = 1). The reported median follow-up across the included studies was 47.7 months (23.7–77.0 months).

### Chemotherapy regimens

Oncological regimens were reported in six studies (*Table 2*). For chemotherapy regimens, in both dCRT and nCRS, cisplatin and 5-fluorouracil (5-FU) was used in three studies, followed by cisplatin and 5-FU/docetaxel in two studies, cisplatin and capecitabine in one study and 5-FU, folinic acid, epidoxorubicin, and cisplatin (FLEP) in one study. For radiotherapy regimens, the total dose delivered ranged from 40.0 to 66.0 Gy for dCRT and 40.0 to 50.4 Gy in nCRS.

**Table 2:** Reporting of treatment regimens for definitive and neoadjuvant chemoradiotherapy of patients with oesophageal squamous cell carcinoma

Study name	Definitive chemoradiotherapy				Neoadjuvant chemoradiotherapy				Time to surgery (days)
	Chemotherapy regimen	Cycles, (*n*)	Radiotherapy dose, (Gy)	Radiotherapy fractions, (*n*)	Chemotherapy regimen	Cycles, (*n*)	Radiotherapy dose, (Gy)	Radiotherapy fractions, (*n*)	
**Randomized clinical trials**									
Stahl 2005^12^	FLEP	3	50–60	25–30	FLEP	3	40	20	NR
Bedenne 2007^13^	CF	5	45 or 66	NR	CF	2	45 or 66	NR	50–60
Park 2019^28^	Cisplatin/capecitabine	2	50.4	28	Cisplatin/capecitabine	2	50.4	28	42–56
**Cohort studies**									
Lee 2003^21^	CF	4	60	40	CF	2	48	40	21–28
Liu 2017^22^	Cisplatin + 5-FU/docetaxel	2	60	NR	Cisplatin + NVB/docetaxel	2	40	20	42
Barbetta 2018^23^	NR	NR	NR	NR	NR	NR	NR	NR	NR
Sheil 2018^24^	NR	NR	NR	NR	CF	NR	40	15	NR
Wang 2019^25^	NR	NR	NR	NR	NR	NR	NR	NR	NR
Duarte 2020^26^	NR	NR	NR	NR	NR	NR	NR	NR	NR
Kamarajah 2020^27^	NR	NR	NR	NR	NR	NR	NR	NR	NR

FLEP, 5-fluorouracil, folinic acid, epidoxorubicin, and cisplatin; CF, cisplatin, 5-fluorouracil; 5-FU, 5-fluorouracil; NVB, navelbine.

### Overall survival

**Fig. 3.**
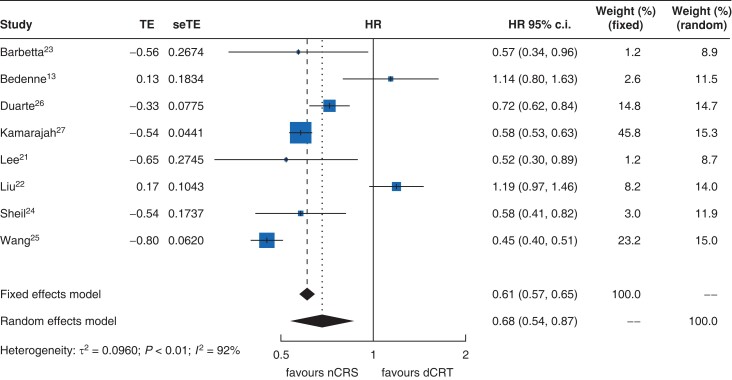
Forest plot for effect of nCRS vs dCRT on overall survival. dCRT, definitive chemoradiotherapy; nCRS, neoadjuvant chemoradiotherapy and surgery; seTE, standard error of treatment estimate; TE, treatment effect

OS was reported in eight studies, of which six reported a survival benefit with nCRS over dCRT; however, two studies did not demonstrate any survival benefit between the two treatment options. In a random-effects meta-analysis, patients who received nCRS demonstrated significantly longer survival compared with those who received dCRT (HR 0.68, 95 per cent c.i. 0.54 to 0.87, *P* < 0.001; *Fig. 3*). There was significant heterogeneity (*I*^2 =^ 92 per cent, *P* < 0.010). Egger regression testing suggested that publication biases were minimal for reporting of OS (*P* = 0.4; *Fig. S1*).

### Disease-free survival

**Fig. 4.**
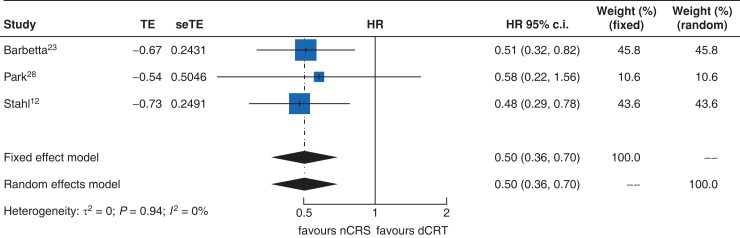
Forest plot of effect of nCRS vs dCRT on disease-free survival. dCRT, definitive chemoradiotherapy; nCRS, neoadjuvant chemoradiotherapy and surgery; seTE, standard error of treatment estimate; TE, treatment effect

DFS was reported in three studies, of which two reported a survival benefit for nCRS over dCRT, and one study reported no difference between groups. In a random-effects meta-analysis, patients who received nCRS demonstrated significantly longer survival than patients who received dCRT (HR 0.50, 95 per cent c.i. 0.36 to 0.70, *P* < 0.001; *Fig. 4*). Heterogeneity was low (*I*^2^ = 0 per cent, *P* = 0.94). Egger regression testing suggested publication biases were minimal for reporting of OS (*P* = 0.4; *Fig. S2*). A sensitivity analysis was performed including only RCTs that demonstrated improvement DFS with nCRS over dCRT (HR 0.50, 95 per cent c.i. 0.32 to 0.77, *P* = 0.002; *Fig. S3*).

### Histopathological outcomes

In patients receiving nCRS, histopathological margin status was reported in five studies. The overall rate of margin-negative resections was 75.1 per cent (263 of 350), ranging from 59.3 to 89.3 per cent. Pathological complete response (pCR) rates were reported in four studies. The overall rate of pCR was 29.1 per cent (90 of 309), ranging from 19.3 to 78.9 per cent. The outlier value of 78.9 per cent was reported by a study that randomized only patients that demonstrated clinical complete response to either dCRT or nCRS^28^.

## Discussion

This review synthesizes presently available high-quality evidence comparing dCRT *versus* nCRS treatment for ESCC. The resulting meta-analysis suggests that nCRS may offer advantages in OS and DFS compared with dCRT; however, it is important to note that there is a significant degree of heterogeneity, which may reflect decision-making processes such as: persistence of tumour after completion of dCRT; recurrence more than 3 months after completion of dCRT (better prognosis); and local toxicity of dCRT (stenosis or perforation). Given the equipoise of current guidelines, and the preferential use of dCRT in some centres, data from this study have crucial implications in the decision-making process by those involved in the care of patients with ESCC globally^29^. There is an urgent need to deliver high-quality research studies as previous trials were conducted more than a decade ago.

The conclusions of this meta-analysis stand in contrast to the two previous RCTs on the topic, which demonstrated no benefit in patients receiving surgery after chemoradiotherapy compared with dCRT alone^12,13^. However, due to underlying quality issues within both these trials, their position of influence on modern treatment guidelines is increasingly being called into question^30^. First, both these trials had small sample sizes and were underpowered for the main outcome. Furthermore, only 66 per cent of patients randomized to surgery in one RCT actually went on to receive surgery^12^. These likely reflect difficulties in randomization and recruitment of patients to these trials^5^. Second, both RCTs lacked reporting surgical quality assurance, including the extent of lymphadenectomy performed. With a reported 90-day mortality of 13 per cent^12^ and 9 per cent^13^, with a majority of deaths occurring in the perioperative interval, this is significantly higher than the perioperative mortality reported by high-volume centres^31–36^ and a recent population-based cohort study reporting a 90-day mortality rate of 8 per cent. Third, with reference to treatments, patients in the surgical arm of the trial by Stahl *et al*. received only 40 Gy of radiation in contrast with 50 Gy for patients in the non-surgical arm. This also highlights the variation internationally and within this study of what radiation dose is considered to be definitive^37^. Further, consideration should be given to strict radiotherapy quality assurance and the finding that salvage oesophagectomy after radiotherapy doses of more than 55 Gy show prohibitive results in terms of morbidity and mortality^38^.

To date, two other meta-analyses^39,40^ have partially compared nCRS with dCRT. One study was published some 10 years ago^39^ and lacked sufficient detail to allow valid meta-analyses between the two treatment modalities, reporting only that nCRS was associated with improved long-term survival (HR 0.81, 95 per cent c.i. 0.70 to 0.95, *P* = 0.008) and R0 rates (OR, HR 1.15, *P* = 0.043) compared with upfront surgery (no comparison between dCRT and nCRS made). Second, another recent network meta-analysis^40^ compared different treatments for oesophageal cancers and demonstrated that nCRS (HR 0.76, 95 per cent c.i. 0.67 to 0.85) and neoadjuvant chemotherapy and surgery (nCS) had significantly longer OS compared with surgery alone. Ranking analysis showed that nCRS with surgery was likely to be the best option in terms of efficacy; however, this review failed to perform stratified analyses for patients with ESCC and adenocarcinoma and to perform comparisons including dCRT.

The present review suggests a survival benefit for patients receiving nCRS, but also highlights the lack of prospective trial data involving modern treatment regimens. It suggests that a high-quality RCT incorporating current practices and treatments may be required to definitively address this question and alter the equipoise offered by current guidelines. The ongoing Swedish-led Neoadjuvant Chemoradiotherapy for Esophageal Squamous Cell Carcinoma *versus* Definitive Chemoradiotherapy with Salvage Surgery as Needed (NEEDS) trial, will aim to provide further evidence on the present topic and is currently in recruitment^41^. The SANO trial^42^ on a related topic, seeks to compare intensive surveillance *versus* oesophagectomy in clinical complete responders with neoadjuvant therapy for oesophageal cancer.

Beyond survival outcomes, there are also few or no data comparing patient-related quality of life. With modern treatments resulting in increasing survival in ESCC overall, there is increasingly a focus on patient choice, acceptability of treatments, and the many factors that affect postoperative quality of life. Previous studies have suggested patients’ willingness to trade a degree of survival benefit for convenience or perceived quality of life^43^, and it is unclear to what degree intensive surveillance regimens after dCRT recommended by trials such as SANO might influence patient choice. Further, while standardization of oncological treatments must be aspired to, the rapid development in this area, such as recent data showing efficacy of nivolumab in oesophageal cancers, means that heterogeneity of treatments will continue to be a problem in considering the evidence for ESCC until an updated RCT is achieved^44^. Finally, the role of surveillance for recurrence following dCRT^42^ and salvage oesophagectomy warrants closer evaluation, especially the high rates of morbidity and mortality in the latter.

There are several limitations to this study that must be considered. First, the selection criteria (adjusted or propensity-matched analysis) to address treatment selection bias and the potential for residual or unmeasured bias remains in retrospective cohort studies. These broadly include: decision-making of dCRT or nCRS by tumour location may not be captured as the former is preferred in patients with proximal cancers because surgery is associated with higher mortality; treatment protocols and preferences, which are varied across hospitals, including a wide range in radiation doses seen within the neoadjuvant group and the definitive chemoradiotherapy groups; and potential differences in approaches to clinical staging and adherence to current staging guidelines were not captured and may have led to heterogeneity and misclassification of clinical stage within the study cohort. Particularly OS data from the included studies demonstrated significant heterogeneity as reported in results, which limits generalizability of these findings; conversely, however, heterogeneity was low in the meta-analysis of DFS, with findings broadly in agreement with OS data.

Meta-regression was not performed owing to the small number of studies included. This study has sought to limit risk of bias by using a methodology to select only high-quality studies, and possibly the dearth of prospective trial data on this important topic means that a high-quality, up-to-date synthesis of available evidence as presented here is necessary and adds value to clinicians. Second, the specific regimens used for chemotherapy were not available in all included studies, especially in registry-based analyses. Therefore, any misclassification was likely to bias survival differences towards the null. As such, it was not possible to make any specific recommendations on the most effective chemoradiotherapy regimen based on any comparative data—although the most up-to-date trial data would seem to suggest the CROSS regimen^6^. Third, recurrence data are not widely reported across the included studies; thus, this present review was not able to analyse the pattern of recurrence following different therapeutic strategies. This would be especially important in the context of cohort studies outside RCTs where follow-up protocols may vary across studies and between nCRS and dCRT. Finally, there is variable reporting of surgical quality control, histopathological outcomes, and salvage oesophagectomies in patients undergoing dCRT, thus precluding a sensitivity analysis comparing patients receiving salvage surgery after dCRT with nCRS. The potential issue of surgical quality control is also highlighted by the high rates of margin positivity reported in the present study, which are significantly higher than benchmarks recommended by the recent ECCG group^45,46^. Though differences in histopathological assessment and patient populations may also play a role^47^, this further draws attention to the need for additional prospective high-quality trial data on this topic.

There remains significant equipoise regarding the optimum radical treatment for ESCC. This meta-analysis suggests a possible benefit for nCRS, compared with dCRT, for both OS and DFS. High-quality prospective trial data are lacking and further research is urgently required to optimize patient care and outcomes

## Supplementary Material

zrac125_Supplementary_Data

## Data Availability

The data that support the findings of this study are available from the corresponding author upon reasonable request.

## References

[1] Ajani JA , D’Amico TA, Bentrem DJ, Chao J, Corvera C, Das P et al Esophageal and esophagogastric junction cancers, version 2.2019, NCCN clinical practice guidelines in oncology. J Natl Compr Canc Netw 2019;17:855–883 31319389 10.6004/jnccn.2019.0033

[2] NICE . Oesophago-gastric Cancer: Assessment and Management in Adults. NICE Guideline NG83. London, UK: NICE, 2018 29400920

[3] National Oesophago-Gastric Cancer Audit . National Oesophago-Gastric Cancer Audit (NOGCA) Annual Report 2020. Leeds: NOGCA, 2021

[4] Kamarajah SK , Phillips AW, Hanna GB, Low D, Markar SR. Definitive chemoradiotherapy compared with neoadjuvant chemoradiotherapy with esophagectomy for locoregional esophageal cancer: national population-based cohort study. Ann Surg 2020;275:526–533 10.1097/SLA.0000000000003941 32865948

[5] Blazeby JM , Strong S, Donovan JL, Wilson C, Hollingworth W, Crosby T et al Feasibility RCT of definitive chemoradiotherapy or chemotherapy and surgery for oesophageal squamous cell cancer. Br J Cancer 2014;111:234–240 24921919 10.1038/bjc.2014.313 PMC4102950

[6] van Hagen P , Hulshof MC, van Lanschot JJ, Steyerberg EW, van Berge Henegouwen MI, Wijnhoven BPL et al Preoperative chemoradiotherapy for esophageal or junctional cancer. N Engl J Med 2012;366:2074–2084 22646630 10.1056/NEJMoa1112088

[7] Sjoquist KM , Burmeister BH, Smithers BM, Zalcberg JR, Simes RJ, Barbour A et al Survival after neoadjuvant chemotherapy or chemoradiotherapy for resectable oesophageal carcinoma: an updated meta-analysis. Lancet Oncol 2011;12:681–692 21684205 10.1016/S1470-2045(11)70142-5

[8] Mariette C , Dahan L, Mornex F, Maillard E, Thomas P-A, Meunier B et al Surgery alone versus chemoradiotherapy followed by surgery for stage I and II esophageal cancer: final analysis of randomized controlled phase III trial FFCD 9901. J Clin Oncol 2014;32:2416–2422 24982463 10.1200/JCO.2013.53.6532

[9] Urba SG , Orringer MB, Turrisi A, Iannettoni M, Forastiere A, Strawderman M. Randomized trial of preoperative chemoradiation versus surgery alone in patients with locoregional esophageal carcinoma. J Clin Oncol 2001;19:305–313 11208820 10.1200/JCO.2001.19.2.305

[10] Lee JL , Park SI, Kim SB, Jung HY, Lee GH, Kim JH et al A single institutional phase III trial of preoperative chemotherapy with hyperfractionation radiotherapy plus surgery versus surgery alone for resectable esophageal squamous cell carcinoma. Ann Oncol 2004;15:947–954 15151953 10.1093/annonc/mdh219

[11] Burmeister BH , Smithers BM, Gebski V, Fitzgerald L, Simes RJ, Devitt P et al Surgery alone versus chemoradiotherapy followed by surgery for resectable cancer of the oesophagus: a randomised controlled phase III trial. Lancet Oncol 2005;6:659–668 16129366 10.1016/S1470-2045(05)70288-6

[12] Stahl M , Stuschke M, Lehmann N, Meyer H-J, Walz MK, Seeber S et al Chemoradiation with and without surgery in patients with locally advanced squamous cell carcinoma of the esophagus. J Clin Oncol 2005;23:2310–2317 15800321 10.1200/JCO.2005.00.034

[13] Bedenne L , Michel P, Bouche O, Milan C, Mariette C, Conroy T et al Chemoradiation followed by surgery compared with chemoradiation alone in squamous cancer of the esophagus: FFCD 9102. J Clin Oncol 2007;25:1160–1168 17401004 10.1200/JCO.2005.04.7118

[14] Liberati A , Altman DG, Tetzlaff J, Mulrow C, Gotzsche PC, Ioannidis JPA et al The PRISMA statement for reporting systematic reviews and meta-analyses of studies that evaluate healthcare interventions: explanation and elaboration. BMJ 2009;339:b2700 19622552 10.1136/bmj.b2700 PMC2714672

[15] Biagi JJ , Raphael MJ, Mackillop WJ, Kong W, King WD, Booth CM. Association between time to initiation of adjuvant chemotherapy and survival in colorectal cancer: a systematic review and meta-analysis. JAMA 2011;305:2335–2342 21642686 10.1001/jama.2011.749

[16] Hanna TP , King WD, Thibodeau S, Jalink M, Paulin GA, Harvey-Jones E et al Mortality due to cancer treatment delay: systematic review and meta-analysis. BMJ 2020;371:m4087 33148535 10.1136/bmj.m4087 PMC7610021

[17] Bero L , Chartres N, Diong J, Fabbri A, Ghersi D, Lam J et al The risk of bias in observational studies of exposures (ROBINS-E) tool: concerns arising from application to observational studies of exposures. Syst Rev 2018;7:242 30577874 10.1186/s13643-018-0915-2 PMC6302384

[18] Higgins JP , Altman DG, Gotzsche PC, Juni P, Moher D, Oxman AD et al The Cochrane Collaboration’s tool for assessing risk of bias in randomised trials. BMJ 2011;343:d5928 22008217 10.1136/bmj.d5928 PMC3196245

[19] Cumpston M , Li T, Page MJ, Chandler J, Welch VA, Higgins JPT et al Updated guidance for trusted systematic reviews: a new edition of the Cochrane handbook for systematic reviews of interventions. Cochrane Database Syst Rev 2019;10:ED000142 31643080 10.1002/14651858.ED000142 PMC10284251

[20] Gottlieb-Vedi E , Kauppila JH, Malietzis G, Nilsson M, Markar SR, Lagergren J. Long-term survival in esophageal cancer after minimally invasive compared with open esophagectomy: a systematic review and meta-analysis. Ann Surg 2019;270:1005–1017 30817355 10.1097/SLA.0000000000003252

[21] Lee JL , Kim SB, Jung HY, Park S-L, Kim D-K, Kim J-H et al Efficacy of neoadjuvant chemoradiotherapy in resectable esophageal squamous cell carcinoma–a single institutional study. Acta Oncol 2003;42:207–217 12852697 10.1080/02841860310010736

[22] Liu S , Qiu B, Luo G, Liang Y, Zheng YZ, Chen ZL et al TNM staging matched-pair comparison of surgery after neoadjuvant chemoradiotherapy, surgery alone and definitive chemoradiotherapy for thoracic esophageal squamous cell carcinoma. J Cancer 2017;8:683–690 28367248 10.7150/jca.17048 PMC5370512

[23] Barbetta A , Hsu M, Tan KS, Stefanova D, Herman K, Adusumilli PS et al Definitive chemoradiotherapy versus neoadjuvant chemoradiotherapy followed by surgery for stage II to III esophageal squamous cell carcinoma. J Thorac Cardiovasc Surg 2018;155:2710–2721 29548582 10.1016/j.jtcvs.2018.01.086 PMC5960990

[24] Sheil F , Donohoe CL, King S, O’Toole D, Cunningham M, Cuffe S et al Outcomes for esophageal squamous cell carcinoma treated with curative intent in a western cohort: should multimodal therapy be the gold standard? World J Surg 2018;42:1485–1495 29075858 10.1007/s00268-017-4289-8

[25] Wang BY , Wu SC, Chen HC, Hung W-H, Lin C-H, Huang C-L et al Survival after neoadjuvant chemoradiotherapy and oesophagectomy versus definitive chemoradiotherapy for patients with oesophageal squamous cell carcinoma. Br J Surg 2019;106:255–262 30395362 10.1002/bjs.11004

[26] Duarte MBO , Pereira EB, Lopes LR, Andreollo NA, Carvalheira JBC. Chemoradiotherapy with or without surgery for esophageal squamous cancer according to hospital volume. JCO Glob Oncol 2020;6:828–836 32552112 10.1200/JGO.19.00360 PMC7328122

[27] Kamarajah SK , Phillips AW, Hanna GB, Low DE, Markar SR. Definitive chemoradiotherapy compared with neoadjuvant chemoradiotherapy with esophagectomy for locoregional esophageal cancer: national population-based cohort study. Ann Surg. 2020;275:526–533 10.1097/SLA.0000000000003941 32865948

[28] Park SR , Yoon DH, Kim JH, Kim Y-H, Kim HR, Lee HJ et al A randomized phase III trial on the role of esophagectomy in complete responders to preoperative chemoradiotherapy for esophageal squamous cell carcinoma (ESOPRESSO). Anticancer Res 2019;39:5123–5133 31519624 10.21873/anticanres.13707

[29] Molena D , Stem M, Blackford AL, Lidor AO. Esophageal cancer treatment is underutilized among elderly patients in the USA. J Gastrointest Surg 2017;21:126–136 27527093 10.1007/s11605-016-3229-5 PMC5637537

[30] Ajani JA , D’Amico TA, Almhanna K, Bentrem DJ, Besh S, Chao J et al Esophageal and esophagogastric junction cancers, version 1.2015. J Natl Compr Canc Netw 2015;13:194–227 25691612 10.6004/jnccn.2015.0028

[31] Portale G , Hagen JA, Peters JH, Chan LS, DeMeester SR, Gandamihardja TAK et al Modern 5-year survival of resectable esophageal adenocarcinoma: single institution experience with 263 patients. J Am Coll Surg 2006;202:588–596 16571425 10.1016/j.jamcollsurg.2005.12.022

[32] Hulscher JB , van Sandick JW, de Boer AG, Wijnhoven BPL, Tijssen JGP, Fockens P et al Extended transthoracic resection compared with limited transhiatal resection for adenocarcinoma of the esophagus. N Engl J Med 2002;347:1662–1669 12444180 10.1056/NEJMoa022343

[33] Munasinghe A , Markar SR, Mamidanna R, Darzi AW, Faiz OD, Hanna GB et al Is it time to centralize high-risk cancer care in the United States? Comparison of outcomes of esophagectomy between England and the United States. Ann Surg 2015;262:79–85 24979602 10.1097/SLA.0000000000000805

[34] Orringer MB , Marshall B, Chang AC, Lee J, Pickens A, Lau CL. Two thousand transhiatal esophagectomies: changing trends, lessons learned. Ann Surg 2007;246:363–372 17717440 10.1097/SLA.0b013e31814697f2 PMC1959358

[35] Oesophago-Gastric Anastomosis Study Group on behalf of the West Midlands Research Collaborative. Rates of anastomotic complications and their management following esophagectomy: results of the oesophago-gastric anastomosis audit (OGAA). Ann Surg 2022;275:e382–e391 33630459 10.1097/SLA.0000000000004649

[36] Low DE , Kuppusamy MK, Alderson D, Cecconello I, Chang AC, Darling G et al Benchmarking complications associated with esophagectomy. Ann Surg 2017;269:291–298 10.1097/SLA.0000000000002611 29206677

[37] Ising MS , Marino K, Trivedi JR, Rojan AA, Dunlap NE, van Berkel V et al Influence of neoadjuvant radiation dose on patients undergoing esophagectomy and survival in locally advanced esophageal cancer. J Gastrointest Surg 2019;23:670–678 30788714 10.1007/s11605-019-04141-z

[38] Kranzfelder M , Schuster T, Geinitz H, Friess H, Buchler P. Meta-analysis of neoadjuvant treatment modalities and definitive non-surgical therapy for oesophageal squamous cell cancer. Br J Surg 2011;98:768–783 21462364 10.1002/bjs.7455

[39] Yuan M , Bao Y, Ma Z, Men Y, Wang Y, Hui Z. The optimal treatment for resectable esophageal cancer: a network meta-analysis of 6168 patients. Front Oncol 2021;11:628706 33777777 10.3389/fonc.2021.628706 PMC7988076

[40] Nilsson M , Trial N. A Study of Chemoradiotherapy Followed by Planned Surgery or by Surveillance and Surgery Only When Needed for Cancer of the Esophagus (NEEDS). https://clinicaltrials.gov/ct2/show/NCT04460352?term=NEEDS&cond=Oesophageal+Cancer&draw=2&rank=5 (accessed 26 August 2021)

[41] Noordman BJ , Shapiro J, Spaander MC, Krishnadath KK, van Laarhoven HWM, van Berge Henegouwen MI et al Accuracy of detecting residual disease after cross neoadjuvant chemoradiotherapy for esophageal cancer (preSANO Trial): rationale and protocol. JMIR Res Protoc 2015;4:e79 26121676 10.2196/resprot.4320 PMC4526968

[42] Thrumurthy SG , Morris JJ, Mughal MM, Ward JB. Discrete-choice preference comparison between patients and doctors for the surgical management of oesophagogastric cancer. Br J Surg 2011;98:1124–1131 21674471 10.1002/bjs.7537

[43] Kelly RJ , Ajani JA, Kuzdzal J, Zander T, Van Cutsem E, Piessen G et al Adjuvant nivolumab in resected esophageal or gastroesophageal junction cancer. N Engl J Med 2021;384:1191–1203 33789008 10.1056/NEJMoa2032125

[44] Low DE , Alderson D, Cecconello I, Chang AC, Darling GE, D’Journo XB et al International consensus on standardization of data collection for complications associated with esophagectomy: esophagectomy complications consensus group (ECCG). Ann Surg 2015;262:286–294 25607756 10.1097/SLA.0000000000001098

[45] Low DE , Kuppusamy MK, Alderson D, Cecconello I, Chang AC, Darling G et al Benchmarking complications associated with esophagectomy. Ann Surg 2019;269:291–298 29206677 10.1097/SLA.0000000000002611

[46] Pucher PH , Green M, Bateman AC, Underwood TJ, Maynard N, Allum WH et al Variation in histopathological assessment and association with surgical quality indicators following oesophagectomy. Br J Surg 2021;108:74–79 33640940 10.1093/bjs/znaa038

