# Supplementary material for: Definitive chemoradiotherapy versus neoadjuvant chemoradiotherapy followed by radical surgery for locally advanced oesophageal squamous cell carcinoma: meta-analysis
Source: BJS Open. 2022 Dec 7;6(6):zrac125. doi: 10.1093/bjsopen/zrac125 (PMC9728519; doi:10.1093/bjsopen/zrac125)
Supplement: zrac125_Supplementary_Data [file zrac125_supplementary_data.docx]

Table S1 Search terms used for systematic review

| 1 | oesophageal.ti,ab. | 68816 |
| --- | --- | --- |
| 2 | esophageal.ti,ab. | 274303 |
| 3 | oesophagus.ti,ab. | 35180 |
| 4 | esophagus.ti,ab. | 137630 |
| 5 | 1 or 2 or 3 or 4 | 426505 |
| 6 | cancer.ti,ab. | 5004438 |
| 7 | adenocarcinoma.ti,ab. | 383253 |
| 8 | squamous cell carcinoma.ti,ab. | 247662 |
| 9 | 6 or 7 or 8 | 5289666 |
| 10 | 5 and 9 | 147323 |
| 11 | oesophageal cancer.ti,ab. | 9592 |
| 12 | esophageal cancer.ti,ab. | 55791 |
| 13 | 10 or 11 or 12 | 147323 |
| 14 | definitive.ti,ab. | 276670 |
| 15 | definite.ti,ab. | 194185 |
| 16 | 14 or 15 | 468156 |
| 17 | neoadjuvant.ti,ab. | 117183 |
| 18 | pre-operative.ti,ab. | 93912 |
| 19 | preoperative.ti,ab. | 719928 |
| 20 | 17 or 18 or 19 | 897927 |
| 21 | chemoradiotherapy.ti,ab. | 56262 |
| 22 | chemoradiation.ti,ab. | 41136 |
| 23 | radiochemotherapy.ti,ab. | 11196 |
| 24 | 21 or 22 or 23 | 101970 |
| 25 | 13 and 16 and 20 and 24 | 1068 |

Table S2 Summary of excluded studies on full text assessment

| **Study Name** | **Reason for Exclusion** |
| --- | --- |
| Lin 2017 | Duplicate data with Wang 2019 |
| Yen 2017 | Duplicate data with Wang 2019 |
| Chen 201 | Duplicate data with Wang 2019 |
| Sakin 2020 | Includes non-curative surgery |
| Nomura 2016 | Inclusion of nCT and not nCRS |
| Nomura 2017 | Inclusion of nCT and not nCRS |
| Murakami 2000 | No inclusion of dCRT cohort |
| Zschaeck 2020 | No inclusion of dCRT cohort |
| Matsuda 2015 | No inclusion of nCRS cohort |
| Takebayashi 2017 | No inclusion of nCRS cohort |
| Li 2019 | No inclusion of nCRS cohort |
| Zhao 2019 | No inclusion of nCRS cohort |
| Faiz 2019 | No overall adjusted HR |
| Burmeister 1995 | No stratified outcomes by SCC |
| Martinez 2001 | No stratified outcomes by SCC |
| Liao 2004 | No stratified outcomes by SCC |
| Javie 2006 | No stratified outcomes by SCC |
| Adams 2007 | No stratified outcomes by SCC |
| Hainsowrth 2007 | No stratified outcomes by SCC |
| Wang 2007 | No stratified outcomes by SCC |
| MacKley 2008 | No stratified outcomes by SCC |
| Morgan 2009 | No stratified outcomes by SCC |
| Smith 2009 | No stratified outcomes by SCC |
| Yamashita 2009 | No stratified outcomes by SCC |
| Berger 2011 | No stratified outcomes by SCC |
| Courrech 2011 | No stratified outcomes by SCC |
| McKenzie 2011 | No stratified outcomes by SCC |
| Salek 2011 | No stratified outcomes by SCC |
| Sridhar 2012 | No stratified outcomes by SCC |
| Piessen 2013 | No stratified outcomes by SCC |
| Saeki 2013 | No stratified outcomes by SCC |
| Taketa 2013 | No stratified outcomes by SCC |
| Karran 2014 | No stratified outcomes by SCC |
| Hategan 2015 | No stratified outcomes by SCC |
| Lin 2015 | No stratified outcomes by SCC |
| Gemici 2016 | No stratified outcomes by SCC |
| Sio 2016 | No stratified outcomes by SCC |
| Yap 2017 | No stratified outcomes by SCC |
| Haefner 2018 | No stratified outcomes by SCC |
| Voncken 2018 | No stratified outcomes by SCC |
| Bhangoo 2020 | No stratified outcomes by SCC |
| Jiang 2020 | No stratified outcomes by SCC |
| Jung 2020 | No stratified outcomes by SCC |
| Mayr 2020 | No stratified outcomes by SCC |
| Pang 2020 | No stratified outcomes by SCC |
| Jiang 2021 | No stratified outcomes by SCC |
| Khalid 2021 | No stratified outcomes by SCC |
| Mishra 2021 | No stratified outcomes by SCC |
| Steber 2021 | No stratified outcomes by SCC |
| Murakami 1998 | Not high validity - No adjusted analyses |
| Delambre 2001 | Not high validity - No adjusted analysis for age, stage and comorbidity |
| Noguchi 2003 | Not high validity - No adjusted analyses |
| Hofheinz 2004 | Not high validity - No adjusted analyses |
| Fujita 2005 | Not high validity - No adjusted analyses |
| Nagata 2006 | Not high validity - No adjusted analyses |
| Hsu 2008 | Not high validity - No adjusted analysis by age and comorbidity |
| Nakamura 2011 | Not high validity - No adjusted analyses |
| Morita 2012 | Not high validity - No adjusted analyses |
| Jingu 2013 | Not high validity - No adjusted analyses |
| Rawat 2013 | Not high validity - No adjusted analyses |
| Kapoor 2016 | Not high validity - No adjusted analyses |
| Chen 2017 | Not high validity - No adjusted analysis for comorbidity |
| Chen 2018 | Not high validity - Not adjusted for co-morbidity |
| Koeter 2018 | Not high validity - Not adjusted for co-morbidity |
| Lin 2018 | Not high validity - No adjusted analyses |
| Munch 2019 | Not high validity - Not adjusted for co-morbidity |
| Schrempf 2019 | Not high validity - No adjusted analyses |
| Shao 2016 | Overlap with NCDB |
| Chapman 2019 | Overlap with NCDB |

Table S3 Summary of Quality assessment of included studies using the ROBINS-E

| **Study Name** | **Confounding Bias** | **Selection Bias** | **Exposure measurement** | **Exposure Deviation** | **Missing data/ Attrition** | **Outcome Measurement Bias** | **Reporting Bias** | **ROBINS-I Score: Overall** |
| --- | --- | --- | --- | --- | --- | --- | --- | --- |
| Lee 2003^21^ | Low | Low | Low | Low | Low | Low | Low | Low |
| Liu 2017^22^ | Low | Low | Low | Low | Low | Low | Low | Low |
| Barbetta 2018^23^ | Low | Low | Low | Low | Low | Low | Low | Low |
| Sheil 2018^24^ | Low | Low | Low | Low | Low | Low | Low | Low |
| Wang 2019^25^ | Low | Low | Low | Low | Low | Low | Low | Low |
| Duarte 2020^26^ | Low | Low | Low | Low | Low | Low | Low | Low |
| Kamarajah 2020^27^ | Low | Low | Low | Low | Low | Low | Low | Low |

Table S4 Risk of bias assessment of included randomised controlled trials

| **Study Name** | **Random sequence generation** | **Allocation concealment** | **Baseline differences** | **Patient blinding** | **Carer blinding** | **Differing from intended treatment** | **Incomplete outcome data** | **Selective outcome reporting** | **Appropriate outcome assessment** | **Overall** |
| --- | --- | --- | --- | --- | --- | --- | --- | --- | --- | --- |
| Stahl 2005^12^ | Low | Unclear | Low | Unclear | Low | Low | Low | Low | Low | Low |
| Bedenne 2007^13^ | Low | Low | Low | Unclear | Low | Low | Low | Low | Low | Low |
| Park 2019^28^ | Low | Unclear | Low | Unclear | Low | Low | Low | Low | Low | HIgh |

Figure S1 Funnel plot on the impact of definitive chemoradiotherapy and neoadjuvant chemoradiotherapy and surgery on overall survival

Figure S2 Funnel plot on the impact of definitive chemoradiotherapy and neoadjuvant chemoradiotherapy and surgery on disease-free survival

Figure S3 Summary of meta-analysis of high-quality studies comparing definitive chemoradiotherapy and neoadjuvant chemoradiotherapy with surgery on disease-free survival in randomised controlled trial only

**References**

1. Ajani JA, D'Amico TA, Bentrem DJ, et al. Esophageal and Esophagogastric Junction Cancers, Version 2.2019, NCCN Clinical Practice Guidelines in Oncology. *J Natl Compr Canc Netw*. Jul 1 2019;17(7):855-883. doi:10.6004/jnccn.2019.0033

2. Association NOC. Oesophago-gastric cancer: Assessment and management in adults. *NICE Guidelines*. 2018;

3. Audit NO-GC. *National Oesophago-Gastric Cancer Audit (NOGCA) Annual Report 2020*. 2020:67. 08/02/2021. Accessed 22/08/2021. <https://www.nogca.org.uk/content/uploads/2021/02/REF217_NOGCA_2020-Annual-Report-FINAL-V2.0.pdf>

4. Kamarajah SK, Phillips AW, Hanna GB, Low D, Markar SR. Definitive Chemoradiotherapy Compared to Neoadjuvant Chemoradiotherapy With Esophagectomy for Locoregional Esophageal Cancer: National Population-Based Cohort Study. *Ann Surg*. May 19 2020;doi:10.1097/SLA.0000000000003941

5. Blazeby JM, Strong S, Donovan JL, et al. Feasibility RCT of definitive chemoradiotherapy or chemotherapy and surgery for oesophageal squamous cell cancer. *Br J Cancer*. Jul 15 2014;111(2):234-40. doi:10.1038/bjc.2014.313

6. van Hagen P, Hulshof MC, van Lanschot JJ, et al. Preoperative chemoradiotherapy for esophageal or junctional cancer. *N Engl J Med*. May 31 2012;366(22):2074-84. doi:10.1056/NEJMoa1112088

7. Sjoquist KM, Burmeister BH, Smithers BM, et al. Survival after neoadjuvant chemotherapy or chemoradiotherapy for resectable oesophageal carcinoma: an updated meta-analysis. *Lancet Oncol*. Jul 2011;12(7):681-92. doi:10.1016/S1470-2045(11)70142-5

8. Mariette C, Dahan L, Mornex F, et al. Surgery alone versus chemoradiotherapy followed by surgery for stage I and II esophageal cancer: final analysis of randomized controlled phase III trial FFCD 9901. *J Clin Oncol*. Aug 10 2014;32(23):2416-22. doi:10.1200/JCO.2013.53.6532

9. Urba SG, Orringer MB, Turrisi A, Iannettoni M, Forastiere A, Strawderman M. Randomized trial of preoperative chemoradiation versus surgery alone in patients with locoregional esophageal carcinoma. *J Clin Oncol*. Jan 15 2001;19(2):305-13. doi:10.1200/JCO.2001.19.2.305

10. Lee JL, Park SI, Kim SB, et al. A single institutional phase III trial of preoperative chemotherapy with hyperfractionation radiotherapy plus surgery versus surgery alone for resectable esophageal squamous cell carcinoma. *Ann Oncol*. Jun 2004;15(6):947-54. doi:10.1093/annonc/mdh219

11. Burmeister BH, Smithers BM, Gebski V, et al. Surgery alone versus chemoradiotherapy followed by surgery for resectable cancer of the oesophagus: a randomised controlled phase III trial. *Lancet Oncol*. Sep 2005;6(9):659-68. doi:10.1016/S1470-2045(05)70288-6

12. Stahl M, Stuschke M, Lehmann N, et al. Chemoradiation with and without surgery in patients with locally advanced squamous cell carcinoma of the esophagus. *J Clin Oncol*. Apr 1 2005;23(10):2310-7. doi:10.1200/JCO.2005.00.034

13. Bedenne L, Michel P, Bouche O, et al. Chemoradiation followed by surgery compared with chemoradiation alone in squamous cancer of the esophagus: FFCD 9102. *J Clin Oncol*. Apr 1 2007;25(10):1160-8. doi:10.1200/JCO.2005.04.7118

14. Liberati A, Altman DG, Tetzlaff J, et al. The PRISMA statement for reporting systematic reviews and meta-analyses of studies that evaluate healthcare interventions: explanation and elaboration. *Bmj*. Jul 21 2009;339:b2700. doi:10.1136/bmj.b2700

15. Biagi JJ, Raphael MJ, Mackillop WJ, Kong W, King WD, Booth CM. Association between time to initiation of adjuvant chemotherapy and survival in colorectal cancer: a systematic review and meta-analysis. *JAMA*. Jun 8 2011;305(22):2335-42. doi:10.1001/jama.2011.749

16. Hanna TP, King WD, Thibodeau S, et al. Mortality due to cancer treatment delay: systematic review and meta-analysis. *BMJ*. Nov 4 2020;371:m4087. doi:10.1136/bmj.m4087

17. Bero L, Chartres N, Diong J, et al. The risk of bias in observational studies of exposures (ROBINS-E) tool: concerns arising from application to observational studies of exposures. *Syst Rev*. Dec 21 2018;7(1):242. doi:10.1186/s13643-018-0915-2

18. Higgins JP, Altman DG, Gotzsche PC, et al. The Cochrane Collaboration's tool for assessing risk of bias in randomised trials. *BMJ*. Oct 18 2011;343:d5928. doi:10.1136/bmj.d5928

19. Cumpston M, Li T, Page MJ, et al. Updated guidance for trusted systematic reviews: a new edition of the Cochrane Handbook for Systematic Reviews of Interventions. *Cochrane Database Syst Rev*. Oct 3 2019;10:ED000142. doi:10.1002/14651858.ED000142

20. Gottlieb-Vedi E, Kauppila JH, Malietzis G, Nilsson M, Markar SR, Lagergren J. Long-term Survival in Esophageal Cancer After Minimally Invasive Compared to Open Esophagectomy: A Systematic Review and Meta-analysis. *Ann Surg*. Feb 21 2019;doi:10.1097/SLA.0000000000003252

21. Lee JL, Kim SB, Jung HY, et al. Efficacy of neoadjuvant chemoradiotherapy in resectable esophageal squamous cell carcinoma--a single institutional study. *Acta Oncol*. 2003;42(3):207-17. doi:10.1080/02841860310010736

22. Liu S, Qiu B, Luo G, et al. TNM Staging Matched-pair Comparison of Surgery After Neoadjuvant Chemoradiotherapy, Surgery Alone and Definitive Chemoradiotherapy for Thoracic Esophageal Squamous Cell Carcinoma. *J Cancer*. 2017;8(4):683-690. doi:10.7150/jca.17048

23. Barbetta A, Hsu M, Tan KS, et al. Definitive chemoradiotherapy versus neoadjuvant chemoradiotherapy followed by surgery for stage II to III esophageal squamous cell carcinoma. *J Thorac Cardiovasc Surg*. Jun 2018;155(6):2710-2721 e3. doi:10.1016/j.jtcvs.2018.01.086

24. Sheil F, Donohoe CL, King S, et al. Outcomes for Esophageal Squamous Cell Carcinoma Treated with Curative Intent in a Western Cohort: Should Multimodal Therapy Be the Gold Standard? *World J Surg*. May 2018;42(5):1485-1495. doi:10.1007/s00268-017-4289-8

25. Wang BY, Wu SC, Chen HC, et al. Survival after neoadjuvant chemoradiotherapy and oesophagectomy versus definitive chemoradiotherapy for patients with oesophageal squamous cell carcinoma. *Br J Surg*. Feb 2019;106(3):255-262. doi:10.1002/bjs.11004

26. Duarte MBO, Pereira EB, Lopes LR, Andreollo NA, Carvalheira JBC. Chemoradiotherapy With or Without Surgery for Esophageal Squamous Cancer According to Hospital Volume. *JCO Glob Oncol*. Jun 2020;6:828-836. doi:10.1200/JGO.19.00360

27. Kamarajah SK, Phillips AW, Hanna GB, Low DE, Markar SR. Definitive Chemoradiotherapy Compared to Neoadjuvant Chemoradiotherapy With Esophagectomy for Locoregional Esophageal Cancer: National Population-Based Cohort Study. *Ann Surg*. 2020;Ahead of Printdoi:10.1097/SLA.0000000000003941

28. Park SR, Yoon DH, Kim JH, et al. A Randomized Phase III Trial on the Role of Esophagectomy in Complete Responders to Preoperative Chemoradiotherapy for Esophageal Squamous Cell Carcinoma (ESOPRESSO). *Anticancer Res*. Sep 2019;39(9):5123-5133. doi:10.21873/anticanres.13707

29. Molena D, Stem M, Blackford AL, Lidor AO. Esophageal Cancer Treatment Is Underutilized Among Elderly Patients in the USA. *J Gastrointest Surg*. Jan 2017;21(1):126-136. doi:10.1007/s11605-016-3229-5

30. Ajani JA, D'Amico TA, Almhanna K, et al. Esophageal and esophagogastric junction cancers, version 1.2015. *J Natl Compr Canc Netw*. Feb 2015;13(2):194-227. doi:10.6004/jnccn.2015.0028

31. Portale G, Hagen JA, Peters JH, et al. Modern 5-year survival of resectable esophageal adenocarcinoma: single institution experience with 263 patients. *J Am Coll Surg*. Apr 2006;202(4):588-96; discussion 596-8. doi:10.1016/j.jamcollsurg.2005.12.022

32. Hulscher JB, van Sandick JW, de Boer AG, et al. Extended transthoracic resection compared with limited transhiatal resection for adenocarcinoma of the esophagus. *N Engl J Med*. Nov 21 2002;347(21):1662-9. doi:10.1056/NEJMoa022343

33. Munasinghe A, Markar SR, Mamidanna R, et al. Is It Time to Centralize High-risk Cancer Care in the United States? Comparison of Outcomes of Esophagectomy Between England and the United States. *Ann Surg*. Jul 2015;262(1):79-85. doi:10.1097/SLA.0000000000000805

34. Orringer MB, Marshall B, Chang AC, Lee J, Pickens A, Lau CL. Two thousand transhiatal esophagectomies: changing trends, lessons learned. *Ann Surg*. Sep 2007;246(3):363-72; discussion 372-4. doi:10.1097/SLA.0b013e31814697f2

35. Griffiths EA, Oesophago-Gastric Anastomosis Study Group on behalf of the West Midlands Research C. Rates of Anastomotic Complications and their Management following Esophagectomy: Results of the Oesophago-Gastric Anastomosis Audit (OGAA). *Ann Surg*. Jan 7 2021;doi:10.1097/SLA.0000000000004649

36. Low DE, Kuppusamy MK, Alderson D, et al. Benchmarking Complications Associated with Esophagectomy. *Ann Surg*. Dec 4 2017;doi:10.1097/SLA.0000000000002611

37. Ising MS, Marino K, Trivedi JR, et al. Influence of Neoadjuvant Radiation Dose on Patients Undergoing Esophagectomy and Survival in Locally Advanced Esophageal Cancer. *J Gastrointest Surg*. Apr 2019;23(4):670-678. doi:10.1007/s11605-019-04141-z

38. Markar S, Gronnier C, Duhamel A, et al. Salvage Surgery After Chemoradiotherapy in the Management of Esophageal Cancer: Is It a Viable Therapeutic Option? *J Clin Oncol*. Nov 20 2015;33(33):3866-73. doi:10.1200/JCO.2014.59.9092

39. Kranzfelder M, Schuster T, Geinitz H, Friess H, Buchler P. Meta-analysis of neoadjuvant treatment modalities and definitive non-surgical therapy for oesophageal squamous cell cancer. *Br J Surg*. Jun 2011;98(6):768-83. doi:10.1002/bjs.7455

40. Yuan M, Bao Y, Ma Z, Men Y, Wang Y, Hui Z. The Optimal Treatment for Resectable Esophageal Cancer: A Network Meta-Analysis of 6168 Patients. *Front Oncol*. 2021;11:628706. doi:10.3389/fonc.2021.628706

41. Nilsson M, Trial N. A Study of Chemoradiotherapy Followed by Planned Surgery or by Surveillance and Surgery Only When Needed for Cancer of the Esophagus (NEEDS). ClinicalTrials.gov. 26/08/2021, 2021. Accessed 26/08/2021, 2021. <https://clinicaltrials.gov/ct2/show/NCT04460352?term=NEEDS&cond=Oesophageal+Cancer&draw=2&rank=5>

42. Noordman BJ, Shapiro J, Spaander MC, et al. Accuracy of Detecting Residual Disease After Cross Neoadjuvant Chemoradiotherapy for Esophageal Cancer (preSANO Trial): Rationale and Protocol. *JMIR Res Protoc*. Jun 29 2015;4(2):e79. doi:10.2196/resprot.4320

43. Thrumurthy SG, Morris JJ, Mughal MM, Ward JB. Discrete-choice preference comparison between patients and doctors for the surgical management of oesophagogastric cancer. *Br J Surg*. Aug 2011;98(8):1124-31; discussion 1132. doi:10.1002/bjs.7537

44. Kelly RJ, Ajani JA, Kuzdzal J, et al. Adjuvant Nivolumab in Resected Esophageal or Gastroesophageal Junction Cancer. *N Engl J Med*. Apr 1 2021;384(13):1191-1203. doi:10.1056/NEJMoa2032125

45. Low DE, Alderson D, Cecconello I, et al. International Consensus on Standardization of Data Collection for Complications Associated With Esophagectomy: Esophagectomy Complications Consensus Group (ECCG). *Ann Surg*. Aug 2015;262(2):286-94. doi:10.1097/SLA.0000000000001098

46. Low DE, Kuppusamy MK, Alderson D, et al. Benchmarking Complications Associated with Esophagectomy. *Ann Surg*. Feb 2019;269(2):291-298. doi:10.1097/SLA.0000000000002611

47. Pucher PH, Green M, Bateman AC, et al. Variation in histopathological assessment and association with surgical quality indicators following oesophagectomy. *Br J Surg*. Jan 27 2021;108(1):74-79. doi:10.1093/bjs/znaa038
